# Neurological injury from virtual reality mishap

**DOI:** 10.1136/bcr-2021-243424

**Published:** 2021-10-12

**Authors:** Natasha Warner, James T Teo

**Affiliations:** 1 Neurorehabilitation, Wellington Hospital, London, UK; 2 Neurology, King's College Hospital NHS Foundation Trust, London, UK; 3 Institute of Psychiatry, Psychology and Neuroscience, Kings College London, London, UK

**Keywords:** neurology, cranial nerves, neurological injury, trauma CNS /PNS

## Abstract

Consumer virtual reality systems are becoming increasingly popular with the increasing availability of devices and gamified technologies. Self-sustained injury risks exist with the use of this technology in the uncontrolled home environment, however, the public awareness of these risks may not be recognised. We present a case of a low- impact virtual reality fall resulting in spinal cord injury, hypoglossal nerve injury, vertebral artery dissection and traumatic brain injury.

## Background

Virtual reality (VR) has been used in commercial applications for many years, it is now becoming more popular for general consumer use. Tech analysts predict that roughly 168 million worldwide will have some form of VR installed by 2023.^1^ The consumer VR systems involve wearing a wireless headset and some may also include handsets or controllers. Safety issues involving these systems have been researched and reported adverse physiological effects which can include cybersickness, a form of visually induced motion sickness that can cause loss of spatial awareness, nausea, dizziness and disorientation. Other short-term effects following VR use have been found and these include; eye soreness and trouble focusing, reduced depth of perception, decreased reaction time, loss of balance and prolonged nausea. Although some research exists surrounding the cybersickness and such as side effects on vision or reaction times, there are no studies regarding more physical risks to domestic users of VR such as trip hazards or repetitive strain injury.^2^ Here, we report a case of a low-impact VR-related fall resulting in spinal cord injury, hypoglossal nerve injury, vertebral artery dissection and post-concussion syndrome/traumatic brain injury. We discuss how this case highlights the risks of this increasingly popular technology in the home environment.

## Case presentation

A normally fit and well, 57-year-old man was admitted to his local hospital with head and neck pain, paraesthesia in the upper limbs, confusion and disorientation following a fall while wearing consumer VR headset. He suffered pretraumatic, peritraumatic and post-traumatic amnesia and had only snippets of recall of the incident. He recalled standing up with the VR headset on and then being in a forward-free-falling scene was presented to him visually. As he was visually presented with an open void that he was falling into, he did not move his hands in any protective position. Collateral history from his family report that he had had an unbraced fall where he fell and hit his forehead on a bannister and then fell face down on the floor, and he lost consciousness for 5 min. On examination, he had two frontal lacerations, he had deviation of the tongue to the right causing dysarthria and swallowing difficulties. On evaluation with the American Spinal Injury Assessment (ASIA), there was crossed impaired sensation and motor impairment at C5 level on left consistent with an incomplete asymmetric central cord (overall level C5 ASIA D). There was no bladder or bowel sphincter dysfunction.

## Investigations

**Figure 1:**
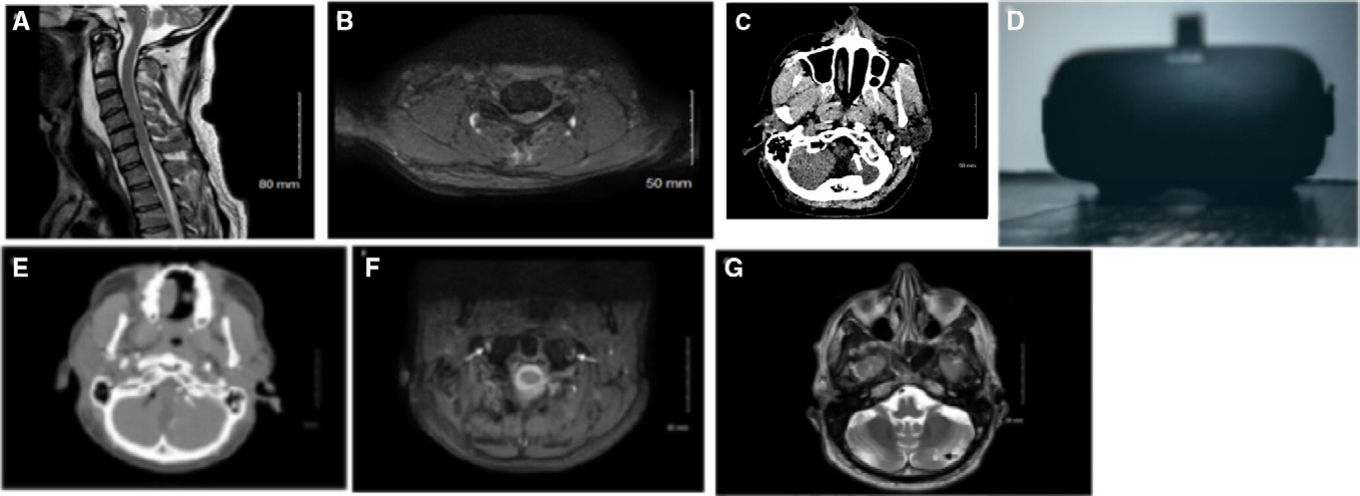
MRI of the cervical spine, sagittal view (A) showing spinal canal stenosis and cord signal change, and axial view at C5–C6 (B). CT head shows hyperdense intracranial right vertebral artery (C) which was subsequently confirmed to be an arterial dissection on CT angiogram (white arrow indicating patent left vertebral artery, black arrow indicating occluded right artery) (E) and MRI dIssection protocol (arrows showing patent left vertebral artery and occluded right vertebral artery) (F). MRI brain showing matured left cerebellar infarct (G). Example of a currently available consumer virtual reality headset (photo by Christine Sandu, via Unsplash) (D).

MRI scan of the cervical spine showed haemorrhagic cord contusion and ligamentous injury at C5 level, acute vertebral body fractures at T3/T4, loss of right vertebral artery flow void (figure 1A, B). A CT scan of head showed bilateral undisplaced occipital condyle fractures, widening of the occipital-atlantal articulation with hyperdense right vertebral artery (figure 1C). CT-intracranial angiogram occlusion of the right vertebral artery (V3 segment and proximal V4) at C1 level with reconstitution prior to joining the patent left vertebral artery (figure 1C, E). This was further confirmed using MRI dissection protocol which showed an evolved occluded right vertebral artery (figure 1F) Limitations of CT intracranial angiography are that vessels can only be imaged at one time which limits the abilty to evaluate flow-related features, it has a lower resolution than catheter angiography making subtle wall changes sometimes difficult to identify and is less accurate in the presence of calcified arteries. Limitations of relying on MRI imaging are that it can not distinguish between intramural thrombus and intramural haematoma or can give rise to false positives caused by high signal intensity of surrounding structures, which can lead to false conclusions.^3^ However, following discussions with radiology, the existing cross-sectional imaging was felt to be sufficient to make this diagnosis without the need for catheter angiogram, MRI angiography or three-dimensional reconstruction imaging. A flexible nasoendoscopy was performed confirmed decreased tongue movement with no structural injury. A later MRI scan of the brain revealed a mature cerebellar hemispheric infarct and no cerebral contusions (figure 1G).

## Treatment

The spinal injuries were evaluated and stabilised non-surgically with a hard ASPEN neck brace for 6 weeks. Antiplatelets was given to reduce further embolic stroke risk from the vertebral artery dissection. He then received further multidisciplinary neurological rehabilitation including spinal physiotherapy, speech and language therapy, and neuropsychology.

## Outcome and follow-up

He made good progress and was ambulant and independent within 2 weeks.

## Discussion

We show in this case the risks of VR systems to self-sustained injuries in home environments. In this case, the mechanism of the injury is low impact yet produced quite extensive distributed injuries; this is due to the individual not using postural safety reflexes to prevent the fall or injury as the visual input over-rode any vestibular or postural input. This case is merely an example of the types of injury a patient can obtain, other physical-related injuries could include a range of musculoskeletal injuries to both bone and soft-tissues, affecting various parts of the body. Immersive visual input provides top-down modulation of many postural reflexes as seen in many physiological and psychophysical experiments^4–8^ and is the basis of some vestibular rehabilitation paradigms.^9^

With increasing availability of consumer-targeted VR devices and gamified technologies like Oculus Rift, HTC Vive, Samsung Gear, Playstation VR, Microsoft Hololens, more injuries are likely in uncontrolled domestic environments unlike controlled environments where these systems are tested and developed. For now, most VR systems remain confined to within short wireless range of a computer workstation, and extension of such technologies outdoors will drive up risk even further.

Some VR devices are also mixed-reality systems which display digital overlays onto real visual scenes (aka augmented reality), and even non-VR systems like smartphones and tablets try to incorporate such interactivity in augmented reality games like Pokemon Go! A recent review showed that even without using VR headsets, virtual experience can distract or reduce inhibitions to hazardous behaviour.^10^

A literature review was commissioned by the department of business, energy and industrial strategy in UK to help understand the safety concerns relating to the use of domestic VR systems, it highlights that the users should be aware of the short-term and longer-term physiological effects after VR use and ways users may mitigate risk.^2^ Although manufacturer health and safety warnings do exist and do advise users to ‘use in safe environments’, advising to clear ‘trip’ hazards and warn about risk of ‘loss of balance’, however it is unknown whether these warnings are in the public awareness and commonly referred to before use.

Self-sustained injuries performed using a consumer device also represent a lacune of liability especially in the context of personal injury claims. It is hard to envisage existing personal injury claims to cover this scenario, and if the rates of such injuries are high, then use of such systems may even impact on health insurance claims. Establishing any liability onto the manufacturer of such systems would likely require a legal test case on whether the user is adequately aware of the risks they have taken on.

**Patient’s perspective**

I fear for casual users of VR headsets. The temptation to simply put on the headset and ‘give it a go’ is almost overwhelming. This is particularly so when, as was the case for me, there is a limited window of time, those with you have used it without incident and the notion that you are a small step away from a life-changing injury does not cross your or anyone else’s mind. The User instruction manual, with its safety warnings. stays on the shelf and you plunge straight into the VR world, or in my case, head first into an immovable object. A fall off a plank into a virtual reality void did not prompt me to cushion my fall with my hands or arms, which stayed casually by my sides as I imitated a felled pine tree. I hope, with the benefit of amazing, effective, insurance funded medical rehabilitation, to make close to a full recovery. However, I understand that if I had been less lucky with my fall, I could have ended up in a wheel chair for the rest of my life.

**Learning points**

- There should be an increased awareness about physical risks and potential injuries associated with domestic Virtual reality (VR).
- Reporting of self-sustained injuries sustained by domestic VR is important to help inform the public and healthcare professionals.
- The lacune of liability especially in the context of personal injury claims.
